# Anomalous Coronary Anatomy with Fistula Diagnosed on Coronary Computed Tomography Angiography

**DOI:** 10.7759/cureus.4403

**Published:** 2019-04-06

**Authors:** Khurram Butt, Ali Agha, Ryan Parente, Joseph Limback, Jeremy R Burt

**Affiliations:** 1 Internal Medicine, Florida Hospital, Orlando, USA; 2 Internal Medicine, University of Texas Health Science Center, Houston, USA; 3 Radiology, Florida Hospital, Orlando, USA

**Keywords:** anomalous coronary artery

## Abstract

Anomalous coronary vasculature is a rare finding among the general population. Identifying such cases is important for preventing adverse outcomes such as sudden cardiac death. We present two rare cases of aberrant coronary anatomy. In Case 1, a 4-year-old male who presented with non-exertional chest pain was found to have anomalous coronary architecture on echocardiogram. Coronary computed tomography angiogram (CCTA) confirmed an anomalous origin of the left coronary artery from the right coronary sinus with a malignant interarterial course and myocardial bridging of the left anterior descending (LAD) artery. The patient underwent a successful surgical correction of the defects. In Case 2, a full-term infant female was born with a hypoplastic right ventricle and pulmonary atresia. CCTA showed a large fistula originating from the coronary sinus on the left that drained into the superior aspect of the mid right ventricular cavity, an anomalous bridge between the left and right atrial appendages, and five fistulous connections between various vessels. The patient was transferred to another facility for cardiac transplant.

## Introduction

Coronary anomalies are rare findings among the general population and are classified according to origin and course, intrinsic coronary anatomy, and termination [1]. Coronary artery fistulas (CAFs) consist of abnormal communication between a coronary artery and another vessel or cardiac chamber [2]. CAFs are observed in 0.002% of the general population. Their prevalence is around 0.08%-0.4% among all congenital heart disease cases and 0.3%-0.8% of catheterization cases [2]. Early diagnosis of coronary anomalies is important for appropriate management and prevention of late symptoms and complications. Coronary angiography via cardiac catheterization remains the gold standard for imaging the coronary arteries; however, the relationship of CAFs to other structures and the origin and course of anomalous coronary arteries may not be apparent. Coronary computed tomography angiogram (CCTA) provides the benefits of better image quality, delineation of anatomical detail, and low dose radiation exposure for detection of coronary artery anomalies in pediatric patients. We present two pediatric cases of coronary artery anomalies identified and managed with the use of CCTA.

## Case presentation

Case 1

**Figure 1:**
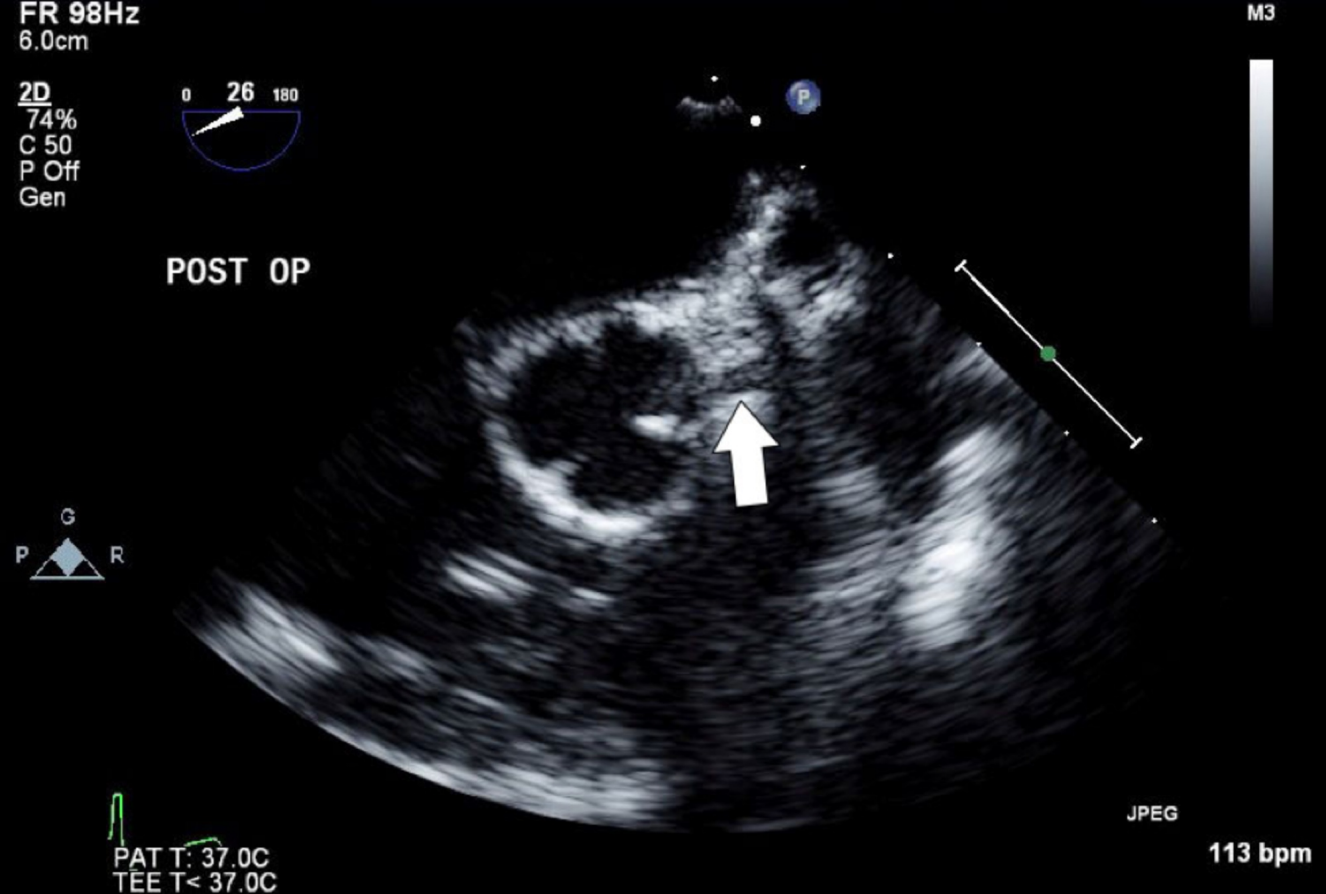
Two-dimensional transesophageal echocardiogram demonstrating the anomalous origin of the left coronary artery from the right coronary sinus (white arrow).

A four-year-old male presented to the pediatric cardiology clinic with a three-week history of episodic chest pain, shortness of breath, diaphoresis, and pallor. Upon presentation, the patient was asymptomatic with an unremarkable physical exam. Office lab work was unremarkable. Electrocardiogram (EKG) showed sinus tachycardia. Echocardiogram showed a possible anomalous origin of the left coronary artery from the right facing sinus (Figure 1).

**Figure 2:**
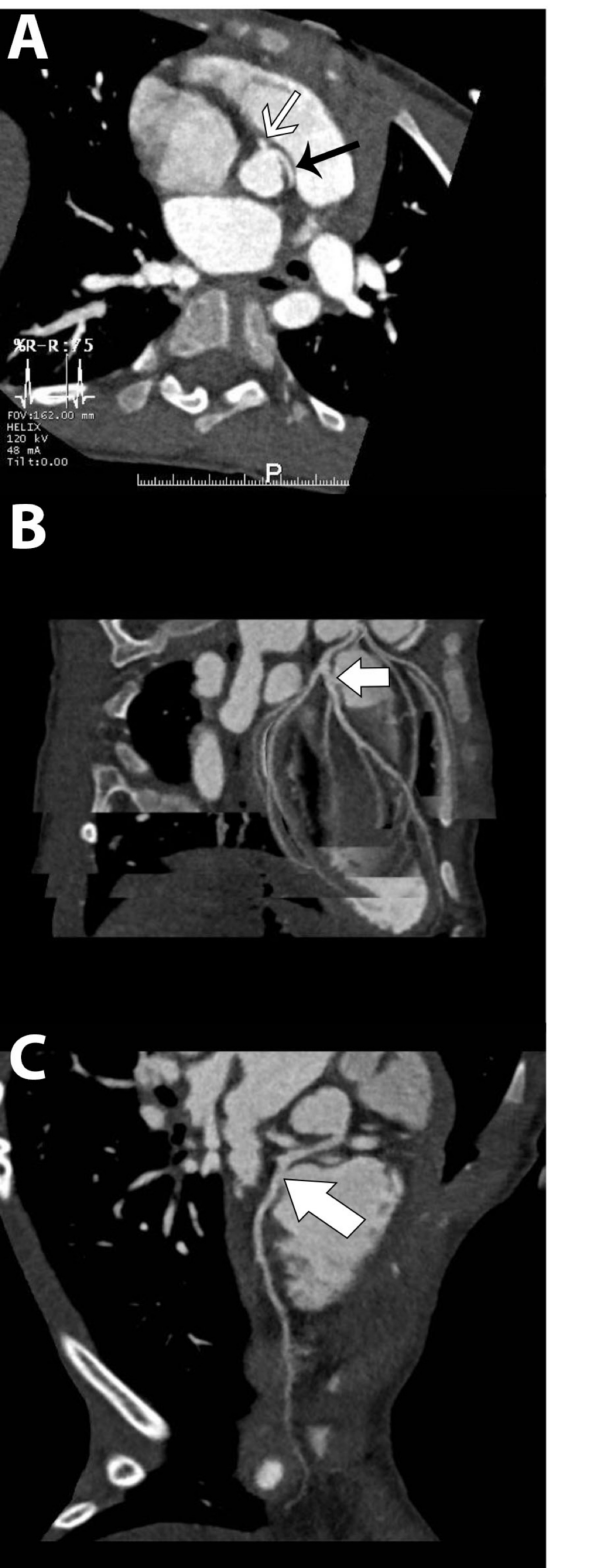
(A) Coronary computed tomography angiogram showing the anomalous origin of the left coronary artery (black arrow) from the right coronary sinus, separate from the right coronary artery (white arrow). (B) Coronary computed tomography angiogram curviplanar reformat showing a fistula from the left anterior descending to the pulmonary trunk (white arrow). (C) Single vessel curviplanar reformat showing left anterior descending-main pulmonary artery fistula (white arrow).

The systolic function and the remainder of his cardiac anatomy were normal. A coronary computed tomography angiogram (CCTA) was performed, which showed both the coronary arteries originating high from the right coronary sinus at the level of the sinotubular junction (Figure 2A). The CCTA was performed on a single source 128-detector row Philips Ingenuity® CT scanner using a low-dose retrospectively ECG-gated helical scan protocol. The heart rate during the scan was 78 beats per minute after the patient received 10 mg of IV esmolol. There was an anomalous origin of the left main coronary artery (LMCA) from the right coronary sinus with a malignant interarterial course between the pulmonary trunk and descending aorta with mild stenosis. It was posited that the LMCA and the right coronary artery (RCA) had a common ostium. There was no evidence that the anomalous LMCA had an intramural course; however, there was a mild narrowing of the interarterial segment with an elliptical shape. This narrowed segment measured approximately 2.3 x 2.0 mm as compared to a more distal normal segment that measured 3.7 x 3.0 mm. Moreover, superficial myocardial bridging was noted with the left anterior descending (LAD) and two significant fistulae were found between the mid and distal LAD and the pulmonary trunk (Figures 2B-2C).

The total dose length product (DLP) of radiation used during CCTA was 122.3 mGy cm and the calculated effective dose (ED) of radiation exposure during CCTA was 3.843 mSv (Table 1).

**Table 1:** Effective dose for computed tomography angiography Conversion factors obtained for computed tomography angiography and calculations were based on the International Commission on Radiological Protection publication 103 recommendations.

Tube voltage (kV)	Conversion factor (mSv/mGy cm)	DLP (mGy cm)	Effective dose (mSv)
80	0.0344	0.8	0.028
100	0.0322	0	0
120	0.0314	121.5	3.815
Total Effective Dose:			3.843

The patient underwent surgical intervention which showed that the LMCA indeed arose from the right coronary sinus and coursed between the aorta and pulmonary artery. The orifice of the LMCA and RCA were so closely positioned together that they made a single coronary artery. The LMCA was mobilized and freed of its attachments followed by unbridging of the LAD. The main pulmonary artery was translocated leftward towards the left pulmonary artery to reduce pressure on the interarterial LMCA. At the end of the surgery, there was excellent flow in all the three coronary arteries.

Case 2

**Figure 3:**
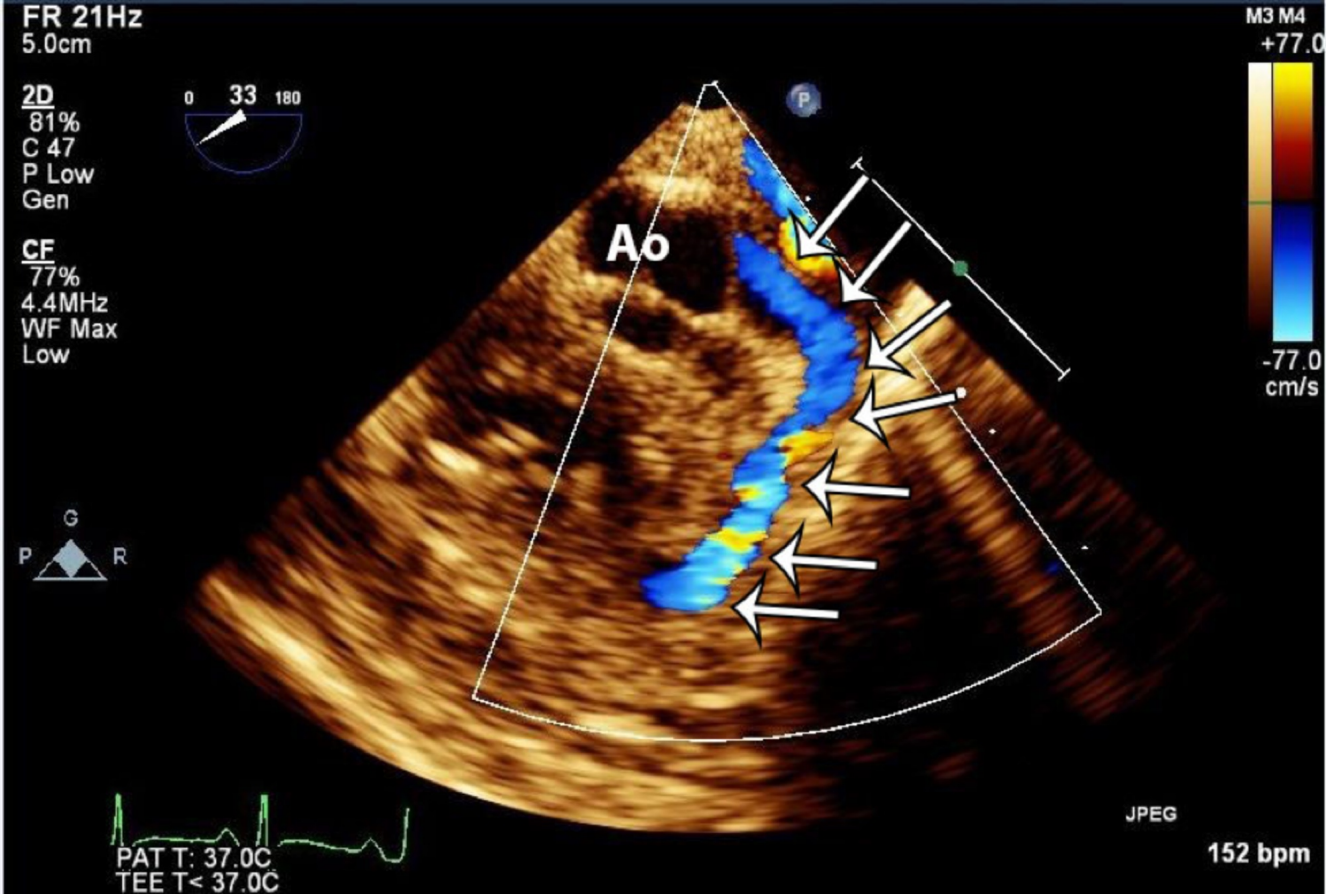
Transesophageal echocardiogram showing a large anomalous coronary artery originating from the left coronary sinus and coursing in the interventricular groove toward the right ventricle (white arrows). Color Doppler shows forward flow in the coronary artery into the right ventricle. Ao: Aortic root

A full-term female infant born with a hypoplastic right ventricle and pulmonary atresia, diagnosed prenatally by ultrasound, was evaluated by pediatric cardiology for further management. EKG at birth was significant for right axis deviation and right ventricular hypertrophy. Echocardiography revealed a large anomalous coronary artery originating from the left coronary sinus and coursing in the interventricular groove toward the right ventricle (Figure 3).

**Figure 4:**
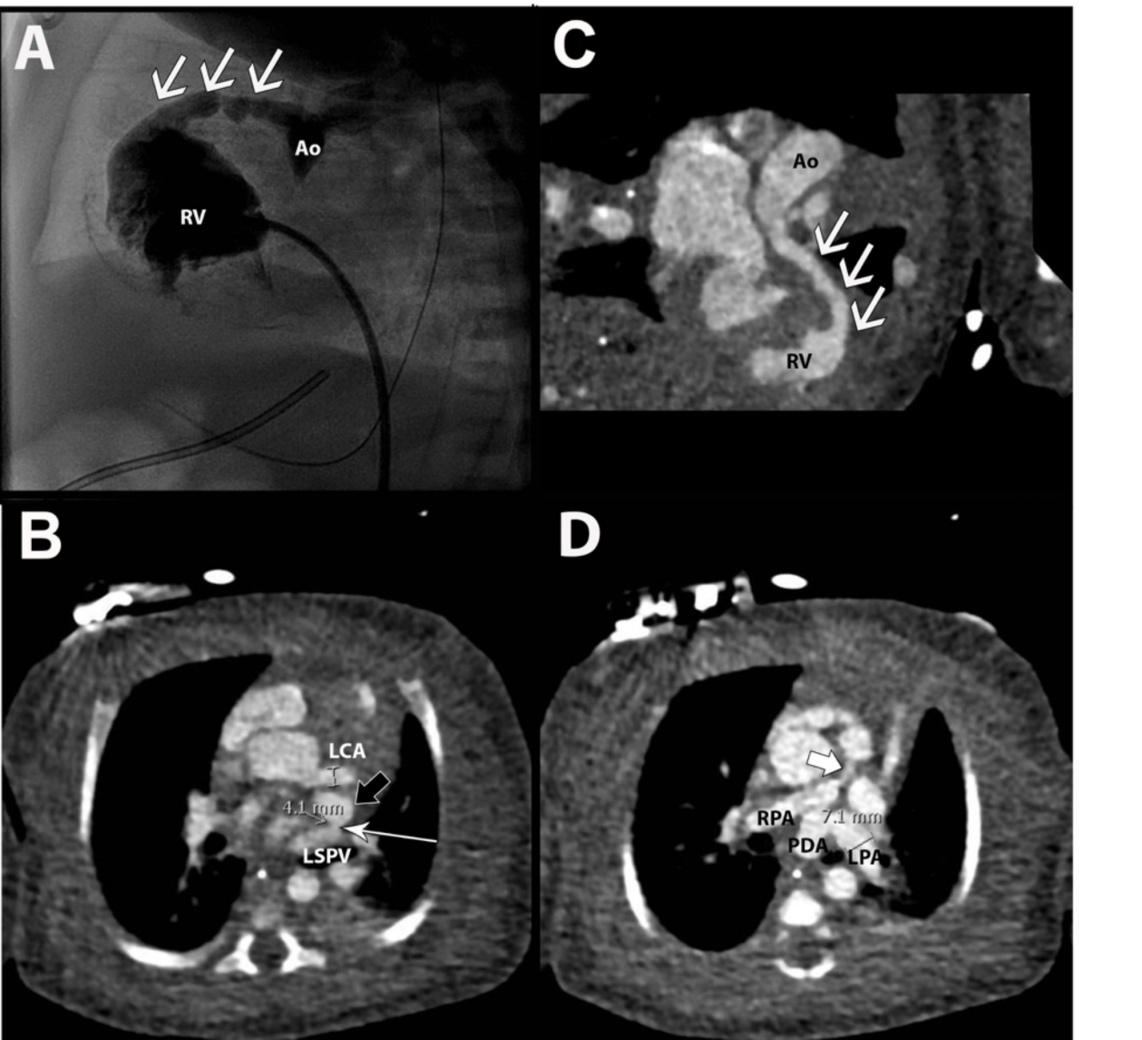
(A) Conventional contrast angiogram with the flow of contrast from the tortuous left anterior descending coronary fistula into the right ventricle (white arrows). (B) Axial coronary computed tomography angiogram demonstrating dilation of the left coronary artery and a fistula (white arrow) from the left superior pulmonary vein to an anomalous venous bridge (black arrow) communicating between the left and right atrial appendages. (C) Curviplanar, maximal intensity projection reformat showing the left anterior descending – right ventricular fistula (white arrow). (D) Axial computed tomography images showing the patent ductus arteriosus, fistula (white arrow), and right and left pulmonary arteries. Ao- aortic root; RV- right ventricle; LCA – left coronary artery; LSPV – left superior pulmonary vein; RPA – right pulmonary artery; LPA – left pulmonary artery; PDA – patent ductus arteriosus.

Cardiothoracic surgery was consulted and a CCTA was ordered to further delineate the coronary anatomy preoperatively. The CCTA was performed on a dual source 64-detector row Siemens® Definition Flash (64 x 2) CT scanner using a low-dose prospectively ECG-gated sequential scan protocol. The heart rate during the scan was 132 beats per minute. Figures 4A-4D demonstrate the anomalous coronary architecture of this patient visualized by coronary angiography and CCTA. The evaluation of coronary anatomy on CCTA showed dilation of the left coronary artery measuring 4.1 mm, and a fistula from the left superior pulmonary vein to an anomalous venous bridge communicating between the left and right atrial appendages. At the level of the proximal-mid LAD, the fistula took an anterior course in the wall of the right ventricular outflow tract and free wall, draining into the superior aspect of the mid right ventricular cavity. There was also a fistulous connection between the LMCA and the left atrial appendage. A large, tortuous patent ductus arteriosus was identified with a distal aneurysm which had limbs draining into the right and left pulmonary arteries. There was no pulmonary trunk. 

**Figure 5:**
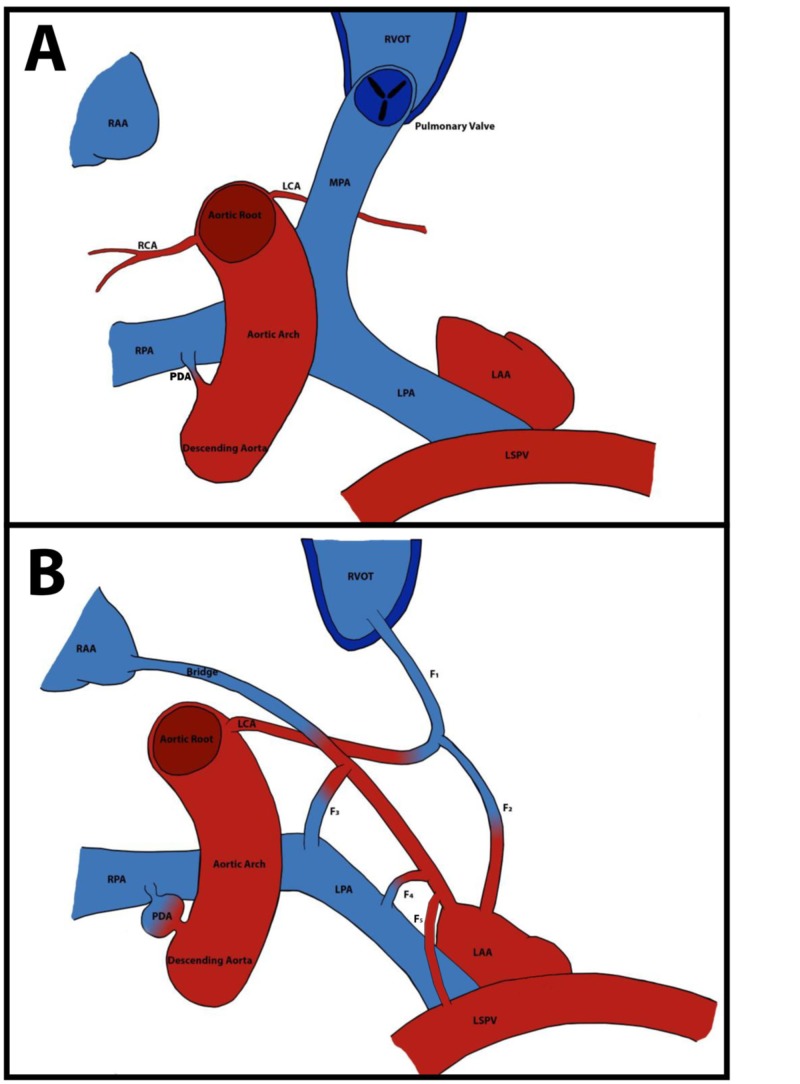
Pictorial representation of normal coronary anatomy (A) compared to the anomalous coronary anatomy (B) seen in Case 2. RAA - right atrial appendage; LAA – left atrial appendage; RVOT - right ventricular outflow tract; RCA – right coronary artery; LCA – left coronary artery; MPA - main pulmonary artery; RPA- right pulmonary artery; LPA – left pulmonary artery; LSPV – left superior pulmonary vein; PDA – patent ductus arteriosus; F1- fistula 1; F2- fistula 2; F3- fistula 3; F4- fistula 4; F5- fistula 5.

Figure 5 depicts normal coronary architecture (Figure 5A) as compared to the anomalous architecture present in this patient (Figure 5B).

Other findings included a large secundum atrial septal defect, mild atrial dilatation, and dilation of the inferior vena cava and the hepatic veins. The total DLP of radiation used during CCTA was 8 mGy cm and the calculated effective dose (ED) of radiation exposure during CCTA was 0.596 mSv (Table 2). Subsequent cardiac catheterization coincided with the findings on the CCTA. The total dose area product (DAP) of radiation used during coronary catheterization was 0.364 Gy cm^2^ and the calculated effective dose (ED) of radiation exposure during coronary catheterization was 0.949 mSv (Table 2).

**Table 2:** Comparison of effective dose for coronary catheterization vs computed tomography angiography Conversion factors obtained for coronary catheterization and computed tomography angiography and calculations were based on the International Commission on Radiological Protection publication 103 recommendations [3-4]

Coronary Catheterization							Computed Tomography Angiography			
Imaging procedure	Conversion factor (mSv/mGy cm)		DAP (Gy cm^2^)	Effective dose (mSv)		Tube voltage (kV)		Conversion factor (mSv/mGy cm)	DLP (mGy cm)	Effective dose (mSv)
Fluoroscopy		2.34	0.254	0.594		80		0.0823	1	0.082
Posteroanterior Angiography		2.2	0.0469	0.103		100		0.0739	6	0.443
Lateral Angiography		4	0.063	0.252		120		0.0706	1	0.071
Total Effective Dose:				0.949		Total Effective Dose:				0.596

Upon surgery, the findings on the CCTA were confirmed. A Gore-Tex shunt was placed from the innominate artery to the right pulmonary artery, followed by pericardial patching of the pulmonary artery and ligation of the patent arterial duct. Fistula repair was also attempted but, owing to the complexity, was not successful. Post-operatively the patient experienced subendocardial ischemia predicted by the EKG findings and rising troponins. A decision was made to transfer the patient to another facility for cardiac transplant.

## Discussion

The presence of coronary artery anomalies are rare findings among the general population and documenting such cases is important for epidemiological monitoring and therapeutic planning. Symptomology and complications depend on the size and location of the coronary anomalies. For example, Case 1 demonstrates a malignant subtype in which the anomalous coronary artery originates from an improper sinus and takes an interatrial course between the aorta and pulmonary artery, which has high risk of sudden cardiac death, especially when the left coronary artery (LCA) originates from the right coronary sinus [1].

Both Case 1 and Case 2 demonstrate multiple CAFs, which are rare findings among the general population. CAF formation is attributed to the persistence of abnormal sinusoidal connections to cardiac structures and can involve connection to a cardiac chamber (coronary-cameral fistula) or to a central venous structure (coronary arteriovenous fistula) [5-7]. Although Case 2 demonstrated a fistula originating from the left coronary sinus, Hu et al. found that 70 % of CAFs in children (mean age 2.9 years) originate from the RCA [8]. The most common sites for fistulous connections are the right ventricle, right atrium, and pulmonary artery [9]. In cases of fistulous insertion into a right-sided heart chamber or systemic venous structure, as in Case 2, there is left to right shunting and volume overload [9]. Proximal to the fistula, aneurysmal dilation may occur, which can lead to early atherosclerosis and, rarely, rupture. A study that evaluated CAFs in adults found that atherosclerosis of coronary arteries occurred mostly near the origin site of CAFs and most aneurysms occurred adjacent to the drainage site [10]. Other rare complications include thrombosis within a fistula, fistula rupture, and pulmonary hypertension [11].

Cardiac catheterization is the gold standard test for diagnosis of coronary anomalies; however, it is limited by its inability to completely opacify the coronary vasculature due to turbulent flow in these vessels, thus, making identification of the three-dimensional course of the artery difficult. CAFs, specifically, drain into low-pressure chambers of the heart and these drainage sites may not be well visualized with conventional angiography because of a significant dilution of the contrast medium [12]. It may also fail to identify aneurysms.

In contrast to conventional methods, CCTA allows for clear depiction of the origin of CAFs, the distal entry site into the receiving chamber/vessel, and proximal and distal coronary anatomy, thereby, facilitating assessment of complex coronary anatomy. CCTA can demonstrate coronary arteries as small as the fifth-order branches. Three-dimensional imaging allows for the determination of the size and location of fistulae at any projection without repeated radiation exposure or additional contrast load for the patient [13]. Virtual reality (VR) imaging is the most common three-dimensional imaging technique and can reliably depict coronary artery anomalies and their relationships to adjacent structures. Curviplanar reformation (CPR) and multiplanar reformation (MPR) are two-dimensional imaging modalities that can supplement VR imaging in showing the shape and size of the CAFs as well as the lumen structure [14]; however, the final diagnosis is based on the raw axial images. Additionally, since most children have high heart rates leading to motion artifacts on imaging, EKG-gating technology can be applied to CCTA to minimize motion artifact by selecting the data from mid-diastole (diastasis) or end-systole (isovolumic relaxation) of each cardiac cycle for image reconstruction.

When choosing a diagnostic imaging modality, diagnostic precision must be weighed against potential risks, which include radiation exposure. Effective dose (ED) must be calculated and compared as a standardized measure of radiation exposure. In Case 2, the effective dose of radiation exposure for conventional catheter-based coronary angiography was 0.949 mSv, which was almost 40% more radiation exposure when compared to CCTA (0.596 mSv). In Case 1, CCTA was shown to have a higher effective dose (3.843 mSv) compared to Case 2, accounted for by the larger size of a four-year-old patient compared to a neonate. Further, in Case 1 CCTA provided accurate and sufficient diagnostic evidence to proceed with therapeutic intervention without the need for cardiac catheterization. Several studies have been conducted in the pediatric population to compare radiation exposure of cardiac catheterization and CCTA. Kawasaki et al. found the mean effective dose for neonatal cardiac catheterization to be 7.7 mSv (range of 0.1-18.4 mSv) [3]. Watson et al. found that among infants less than 12 months of age, the median effective dose for the CTA group was 0.73 mSv (range 0.43-7 mSv) compared with 13.4 mSv (range 4.85-74.6 mSv) for the catheterization group (*p* < 0.0001) [4].

Echocardiography and magnetic resonance angiography can also be used for the evaluation of coronary artery anomalies. Echocardiography is a good initial test for detection of coronary anomalies; however, it has limited efficacy in the visualization of complex anatomy [15-18]. In contrast, magnetic resonance angiography has high resolution with no radiation or iodinated contrast exposure. However, it has a long acquisition time, generally lower spatial resolution, and a contrast-to-noise ratio inferior to that of CCTA [18-19], especially for evaluation of distal coronary arteries and extracardiac structures [20]. Further, this method has limited use for patients with claustrophobia, pacemakers, surgical clips, and arrhythmias.

## Conclusions

Overall, CCTA demonstrates superiority to cardiac catheterization with respect to its lower exposure to radiation and high diagnostic precision. Furthermore, CCTA is less time consuming and bypasses the procedural risks and complications associated with catheterization. Based on the multiple benefits described and low radiation exposure, CCTA may be more advantageous than conventional coronary angiography for accurate diagnosis and therapeutic planning of coronary artery anomalies in pediatric patients.
